# Oral microbiota reveals signs of acculturation in Mexican American women

**DOI:** 10.1371/journal.pone.0194100

**Published:** 2018-04-25

**Authors:** Kristi L. Hoffman, Diane S. Hutchinson, Jerry Fowler, Daniel P. Smith, Nadim J. Ajami, Hua Zhao, Paul Scheet, Wong-Ho Chow, Joseph F. Petrosino, Carrie R. Daniel

**Affiliations:** 1 Department of Epidemiology, Division of Cancer Prevention and Population Sciences, The University of Texas MD Anderson Cancer Center, Houston, Texas, United States of America; 2 Alkek Center for Metagenomics and Microbiome Research, Department of Molecular Virology and Microbiology, Baylor College of Medicine, Houston, Texas, United States of America; Case Western Reserve University, UNITED STATES

## Abstract

The oral microbiome has been linked to a number of chronic inflammatory conditions, including obesity, diabetes, periodontitis, and cancers of the stomach and liver. These conditions disproportionately affect Mexican American women, yet few studies have examined the oral microbiota in this at-risk group. We characterized the 16S rDNA oral microbiome in 369 non-smoking women enrolled in the MD Anderson *Mano a Mano* Mexican American Cohort Study. Lower bacterial diversity, a potential indicator of oral health, was associated with increased age and length of US residency among recent immigrants. Grouping women by overarching bacterial community type (e.g., “*Streptococcus*,” “*Fusobacterium*,” and “*Prevotella*” clusters), we observed differences across a number of acculturation-related variables, including nativity, age at immigration, time in the US, country of longest residence, and a multi-dimensional acculturation scale. Participants in the cluster typified by higher abundance of *Streptococcus spp*. exhibited the lowest bacterial diversity and appeared the most acculturated as compared to women in the “*Prevotella”* group. Computationally-predicted functional analysis suggested the *Streptococcus*-dominated bacterial community had greater potential for carbohydrate metabolism while biosynthesis of essential amino acids and nitrogen metabolism prevailed among the *Prevotella*-high group. Findings suggest immigration and adaption to life in the US, a well-established mediator of disease risk, is associated with differences in oral microbial profiles in Mexican American women. These results warrant further investigation into the joint and modifying effects of acculturation and oral bacteria on the health of Mexican American women and other immigrant populations. The oral microbiome presents an easily accessible biomarker of disease risk, spanning biological, behavioral, and environmental factors.

## Introduction

Hispanics/Latinos comprise nearly 18% of the US population and more than 35% of the state populations of Texas, New Mexico, and California [1]. The majority of US Latinos are first and second generation immigrants of Mexican origin [2,3]. Nearly half of Mexican American women are obese, and 27% do not have health insurance [4]. Hence, it is not surprising that Mexican American women suffer from disproportionate rates of a number of prominent health conditions related to obesity and/or chronic infection, including diabetes [4], uncontrolled hypertension [4], and cancers of the uterine cervix, stomach, and liver [5,6]. Acculturation, the process by which immigrants adapt to a new culture through changes in beliefs and behaviors, is associated with increased rates of obesity [7–9], diabetes [10,11], and cardiovascular risk factors [12,13]—all of which contribute to health disparities and chronic disease burden in this population.

The oral microbiome and its role in health and disease is a rapidly progressing research area with the potential to transform approaches to major public health problems currently facing Mexican American women; however, few studies have investigated the oral microbiota in this at-risk group. Emerging evidence links poor oral health to increased risk of cardiovascular disease [14,15], rheumatoid arthritis [16], and several cancers [17]. While the specific role of the oral microbiota remains unclear, research suggests bacteria in the oral cavity may influence disease by modulating inflammation and genome stability [18]. The oral microbiome is relatively stable throughout adult life [19–21], but inter- and intra-individual variations have been linked to tobacco use [22], diet [23], and oral hygiene [24–26]. Differences by geography and culture, which may reflect variation in diet, host genetics, or other factors, have also been observed [27–29]. Hence, the oral microbiome is poised to become a promising and easily accessible biomarker, potentially reflecting the intersection of biological, behavioral, and environmental risk factors that we cannot comprehensively measure or quantify in human epidemiologic studies.

We characterized the 16S oral microbiome in a group of 369 first and second generation Mexican American women enrolled in the MD Anderson *Mano a Mano* Mexican American Cohort Study (MACS) [30], a large prospective study of the genetic, social, and behavioral risk factors that contribute to cancer and chronic disease risk in the Mexican American population of Houston, Texas. We investigated the oral microbiota with respect to baseline demographic, acculturation, and health-related risk factors. Understanding these relationships will improve our knowledge of the oral microbiome and its potential as a biomarker of exposures and/or subsequent disease in Mexican American women.

## Materials and methods

### Study population and sample collection

The MD Anderson MACS cohort is an ongoing (enrollment 2001-) prospective, population-based study of predominantly low income, first generation immigrants of Mexican origin residing in the greater Houston metropolitan area [30]. All procedures in the current study as well as the parent Cohort were approved by the University of Texas MD Anderson Institutional Review Board and carried out in accordance with the appropriate regulations. Cohort participants provided written informed consent in the language of their choosing, English or Spanish. Upon enrollment, a baseline in-home interview consisting of questionnaires, measurement of height and weight, and biospecimen collection was conducted. Linguistic acculturation was measured using eight items from the Bidimensional Acculturation Scale for Hispanics [31]. Specifically, participants were asked how frequently they spoke, read, watched television programs, and listened to radio programs in English and Spanish. Responses were scored for each dimension. Over 85% of participants in the current study were classified as having high Hispanic acculturation; hence, analysis was limited to acculturation in the English dimension only. A query on food acculturation from the Cultural Life Style Inventory [32] was added to the baseline questionnaire in 2006. Physical activity was measured using an instrument derived from the California Teachers Study survey [33]. Further details of the MACS study can be found elsewhere [30].

For the current study, we randomly selected 375 adult women participating in the MACS cohort who met the following criteria: never smoker, not currently pregnant, and age ≥20 years at enrollment. Selection was further refined to those with complete data across key variables of interest. Current and former tobacco users were excluded to focus on associations with other, less known factors. Participants in the current study were characteristically similar to all women in the overall cohort [30], over 80% of whom are never smokers. Participant demographic data are available in S1 File.

Oral mouthwash samples, originally collected and processed at baseline for the primary purpose of human genetic association studies, were used for microbial analysis. Briefly, participants were asked to swish an alcohol-based mouthwash for 30 seconds, after which samples were collected and transported on ice to the laboratory where cellular content was isolated and resuspended in TE buffer. Samples were frozen at -80°C, where they remained until the point of processing for microbial 16S rDNA sequencing.

### Laboratory methods/16S sequencing

DNA was extracted from the oral mouthwash cellular matter using the MoBio PowerSoil DNA isolation kit following manufacturer’s instructions. 16S rDNA sequencing was performed using Illumina MiSeq with barcoded primers targeting the V4 region: GGACTACHVGGGTWTCTAAT and GTGCCAGCMGCCGCGGTAA [34]. Raw sequences were merged and quality filtered using USEARCH [35]. Parameters for merging included minimum overlap of 50 base pairs, zero mismatches, and truncation quality value of 5. Quality filtering allowed for a maximum expected error rate of 0.05. Illumina PhiX control sequences were removed with Bowtie2 [36]. All remaining sequences were subsequently clustered into operational taxonomic units (OTUs), with chimera removal using UPARSE [37]. Taxonomy assignment was performed closed-reference against the SILVA database (release 123) at 97% identity, resulting in 7,083,883 total reads (median 18,190 reads/sample). Samples that produced fewer than 4,000 reads were excluded from subsequent analysis. The remaining samples (n = 369) were rarefied to 7,600 reads/sample. UPARSE centroid OTU sequences were queried via Basic Local Alignment Search Tool (BLAST) [38] to identify likely representative bacterial species. A rarefied OTU table with the associated centroid sequences is available in S2 File. Bacterial functional capabilities were imputed using Tax4Fun [39], an algorithm using phylogenetic relationships to predict gene content, ultimately assigning functional pathways using the Kyoto Encyclopedia of Genes and Genomes [40,41].

### Statistical analysis

Bacterial alpha diversity was measured using observed OTU (total number of unique OTUs per sample), Chao1 index, and Shannon diversity index. Differences in alpha diversity by demographic and health behavior variables were analyzed by ANOVA or linear regression. Beta diversity was measured using Bray-Curtis dissimilarity distance and analyzed via permutational multivariate analysis of variance. Sparse Correlations for Compositional data (SparCC) [42], which accounts for the compositional nature of 16S sequencing, was used to identify bacterial co-occurrence using false discovery rate Q = 0.01. Microbiota-derived clustering was performed using Dirichlet multinomial mixtures (DMM) modeling [43]. To ensure cluster consistency, the DMM algorithm was repeated in 20 separate datasets at various levels of rarefaction (3,000–15,000 reads/sample). Seventy percent of datasets indicated three as the optimum number of clusters; thus, we generated three clusters for the dataset analyzed here. Differences in demographic risk factors across bacterial cluster were tabulated via contingency table and assessed via Pearson’s chi-square. Differentially abundant bacterial taxa and putative functional content of clusters were determined using Linear Discriminant Analysis (LDA) Effect Size (LEfSe) [44], applying the one-against-all strategy with a minimum logarithmic LDA score (i.e., biomarker effect size) of 2.5 and α = 1E-5. Statistical analyses were performed using SAGE Microbiome Explorer [45], R [46], or STATA 14 (StataCorp LP; College Station, TX), as appropriate.

## Results

### Participant characteristics

Baseline characteristics of the 369 non-smoking Mexican American women are detailed in Table 1. Median age was 39 years (range 20–78 years; birth years 1929–1989), more than 75% were currently married, and 50% had less than a high school education. The majority (80%) were born in Mexico, but of these, 52% had lived in the US for 15 years or more (immigration year range 1959–2009 for all participants). Rates of overweight and obesity were 36% and 47%, respectively—similar to those of the overall MACS cohort [30] and Mexican American women nationally [4].

**Table 1:** Observed OTUs by demographic characteristics in Mexican American women (N = 369 unless otherwise indicated).

		Unadjusted		Adjusted for age		Adjusted for age & education	
	N (%)^a^	Mean (SE)	*P*	Mean (SE)	*P*	Mean (SE)	*P*
Age (years)							
20–29	47 (12.7)	106.1 (3.9)					
30–39	148 (40.1)	98.3 (2.2)					
40–49	94 (25.5)	98.5 (2.8)	<0.01				
≥50	80 (21.7)	87.3 (3.0)				
Education level							
< High school	183 (49.6)	98.3 (2.0)	0.62	99.0 (2.0)	0.32		
High school diploma or equivalent	87 (23.6)	95.1 (2.9)		94.3 (2.9)			
> High school	97 (26.3)	95.9 (2.8)		95.4 (2.7)			
Marital status							
Married	279 (75.6)	97.6 (1.6)	0.31	96.9 (1.6)	0.91	96.2 (1.7)	0.99
Not married	89 (24.1)	94.3 (2.9)		96.5 (2.9)		96.1 (2.9)	
Country of birth							
Mexico	294 (79.7)	97.7 (1.6)	0.28	97.6 (1.6)	0.33	96.8 (1.7)	0.48
US	75 (20.3)	93.9 (3.1)		94.3 (3.1)		94.3 (3.1)	
Country of longest residence							
Mexico	216 (58.5)	99.7 (1.8)	0.02	98.9 (1.8)	0.11	97.9 (2.0)	0.21
US	153 (41.5)	93.0 (2.2)		94.3 (2.2)		94.2 (2.2)	
Age of immigration (years)^b^							
0–18	71 (23.7)	92.1 (3.2)	0.27	88.6 (3.3)	0.02	88.5 (3.4)	0.02
19–24	95 (31.7)	98.5 (2.8)		96.5 (2.8)		96.1 (2.9)	
25–29	60 (20.0)	100.9 (3.5)		102.4 (3.5)		101.8 (3.6)	
≥30	74 (24.7)	98.2 (3.2)		102.8 (3.4)		102.2 (3.5)	
Time in US (years)^b^							
<5	23 (7.7)	110.9 (5.6)	0.01	110.0 (5.8)	0.10	109.3 (5.9)	0.14
5–9	48 (16.0)	99.5 (3.9)		98.9 (4.0)		98.6 (4.0)	
10–14	72 (24.0)	101.4 (3.2)		101.1 (3.2)		100.5 (3.4)	
15–19	70 (23.3)	97.0 (3.2)		96.6 (3.3)		95.9 (3.4)	
20–24	28 (9.3)	93.0 (5.1)		93.5 (5.1)		93.3 (5.2)	
≥25	59 (19.7)	88.1 (3.5)		89.5 (4.0)		89.5 (4.1)	
English acculturation score							
1–1.75	152 (41.2)	99.1 (2.2)	0.20	99.6 (2.2)	0.15	98.6 (2.5)	0.27
2–2.75	109 (29.5)	98.0 (2.6)		97.5 (2.6)		97.6 (2.6)	
3–4	106 (28.7)	93.1 (2.6)		93.0 (2.6)		92.9 (2.6)	
Food acculturation^c^							
Only Mexican foods	55 (16.5)	101.2 (3.5)	0.14	100.9 (3.4)	0.16	99.4 (3.6)	0.28
Mostly Mexican foods	117 (35.1)	97.4 (2.4)		97.3 (2.4)		96.7 (2.4)	
Mix /Mostly American/Other	161 (48.3)	93.5 (2.0)		93.6 (2.0)		93.3 (2.0)	
History of alcohol consumption							
No	310 (84.0)	97.9 (1.5)	0.12	98.0 (1.5)	0.08	97.3 (1.6)	0.12
Yes	59 (16.0)	91.9 (3.5)		91.4 (3.5)		91.3 (3.5)	
History of farm work							
No	284 (77.0)	97.5 (1.6)	0.49	97.2 (1.6)	0.70	96.6 (1.6)	0.61
Yes	85 (23.0)	95.2 (3.0)		96.0 (2.9)		94.9 (3.0)	
BMI (kg/m^2^)							
Lean (<25)	63 (17.1)	101.0 (3.4)	0.34	99.6 (3.4)	0.33	99.5 (3.4)	0.37
Overweight (25–29)	132 (35.8)	96.0 (2.4)		96.2 (2.3)		95.4 (2.4)	
Obese class I (30–34)	104 (28.2)	94.1 (2.7)		93.9 (2.6)		93.2 (2.7)	
Obese class II+ (≥35)	70 (19.0)	99.4 (3.3)		100.6 (3.2)		99.2 (3.3)	
Physical activity level (tertiles)							
Light	132 (35.8)	96.3 (2.4)		97.3 (2.3)		96.2 (2.4)	
Moderate	145 (39.3)	97.7 (2.3)	0.83	97.4 (2.2)	0.72		96.7 (2.3)	0.74
Heavy	88 (23.9)	95.7 (2.9)		94.7 (2.9)		94.0 (2.9)	

Abbreviations: BMI, body mass index; OTU, operational taxonomic unit; SE, standard error.

^a^ Totals may not add up to 100% due to missing responses in <3% of participants.

^b^ Those not born & raised in US; N = 300.

^c^ Variable not assessed prior to 2006; N = 333 for 2006–2011 enrollment.

### A core oral microbiome

**Fig 1:**
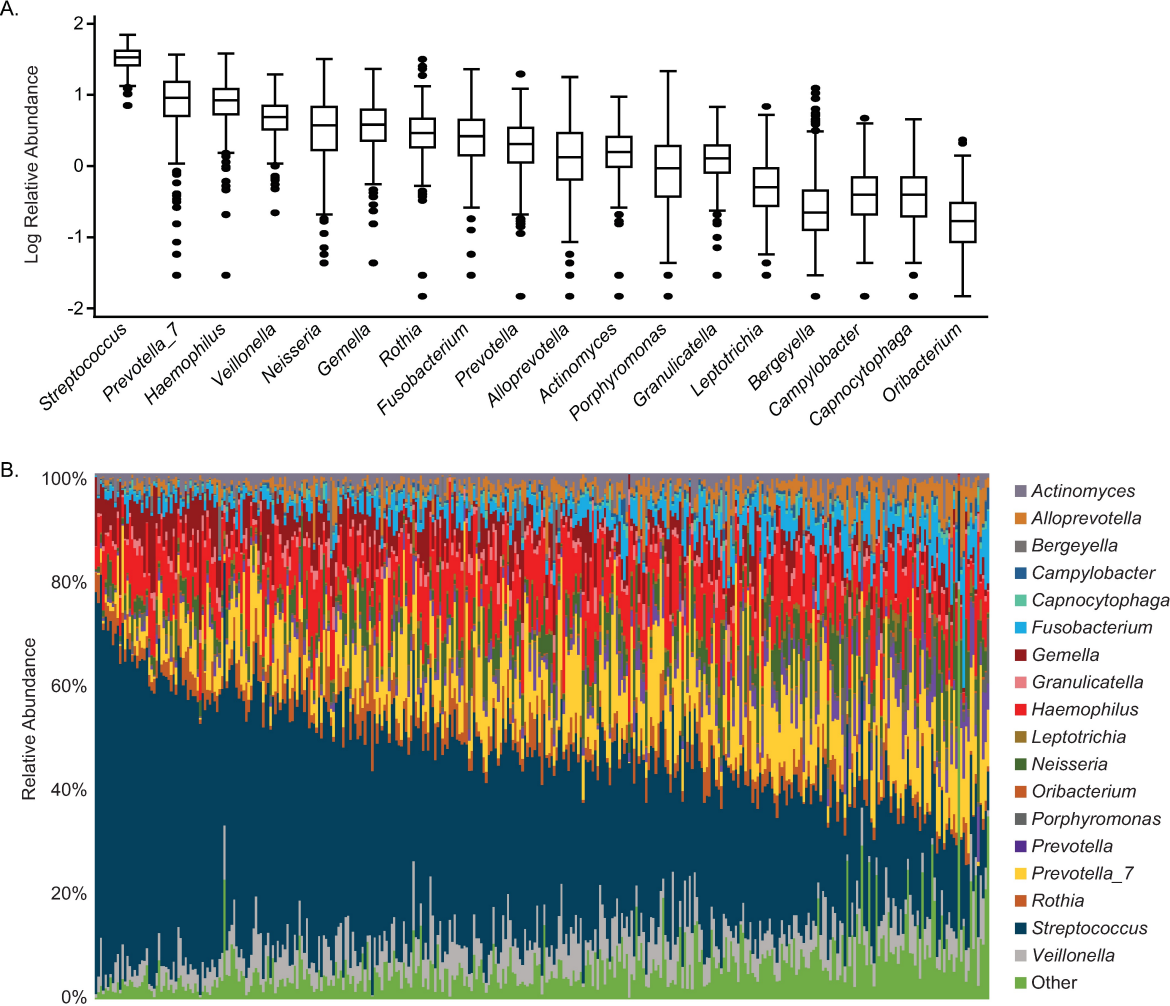
Core oral genera of Mexican American women (Houston, TX, 2004–2011) exhibit wide variation in relative abundance. (A) Box plots indicate log relative abundance of 18 core taxa, defined as those genera detected in ≥ 98% of study subjects. (B) Stacked bar plot shows the contribution of all core genera across each study participant.

A total of 511 unique OTUs, a proxy for bacterial species, were detected in participant mouthwash samples using 16S rRNA gene sequencing (average 97 OTUs/sample). Assigning OTUs at the genus level, we identified a core oral microbiome defined by 18 genera detected in 98% or more of samples (Fig 1A). Consistent with other large-scale studies of the oral microbiota [28,29,47], *Streptococcus* was the most abundant genus, with mean (SD) relative abundance of 37% (12%). *Prevotella_7* (11%), *Haemophilus* (10%), *Veillonella* (6%), and *Neisseria* (5%) completed the top five. Other core bacteria included, in order of relative abundance, *Gemella*, *Rothia*, *Fusobacterium*, *Prevotella*, *Alloprevotella*, *Actinomyces*, *Porphyromonas*, *Granulicatella*, *Leptotrichia*, *Bergeyella*, *Campylobacter*, *Capnocytophaga*, and *Oribacterium*. Current classification using the SILVA rRNA gene database differentiates *Prevotella* from *Prevotella_7* by OTU content. Taxa abundance was marked by considerable inter-individual variability (Fig 1A and 1B), with core genera accounting for 65.2%-99.7% of sequences/sample in all but one person.

### Host-microbiome relationships: Bacterial alpha diversity

We evaluated bacterial alpha diversity with respect to several key demographic and health behavior variables. Notably, age was inversely associated with taxonomic richness as measured by observed OTU and Chao index (*P*<0.01) (Table 1 and S1 Table). This relationship appeared more pronounced in those having lived longer in the US than Mexico, regardless of country of birth (S1 Fig). However, the magnitudes of correlation were modest (observed OTU, r = -0.28; Chao, r = -0.32) and not observed with Shannon diversity (S2 Table and S1 Fig).

Among women born and/or raised in Mexico, taxonomic richness was also inversely correlated with years lived in the US (Table 1). Adjustment for age at sample collection attenuated this association but strengthened the relationship between observed OTU and age at immigration. Taxonomic richness was 16% higher among those immigrating at or after age 30 compared with those arriving at age 18 or prior (88.6 vs 102.8 OTUs, *P* = 0.02). Additional adjustment for educational attainment, a proxy for socioeconomic status, did not meaningfully change these relationships. Of the remaining variables examined, including educational attainment, marital status, country of birth, history of farm work, alcohol consumption, body mass index (BMI), physical activity, and two acculturation metrics representing English linguistic acculturation [31] and the type of food typically eaten at home [32], none were associated with alpha diversity in univariate or multivariate analyses.

### Host-microbiome relationships: Bacterial communities

**Fig 2:**
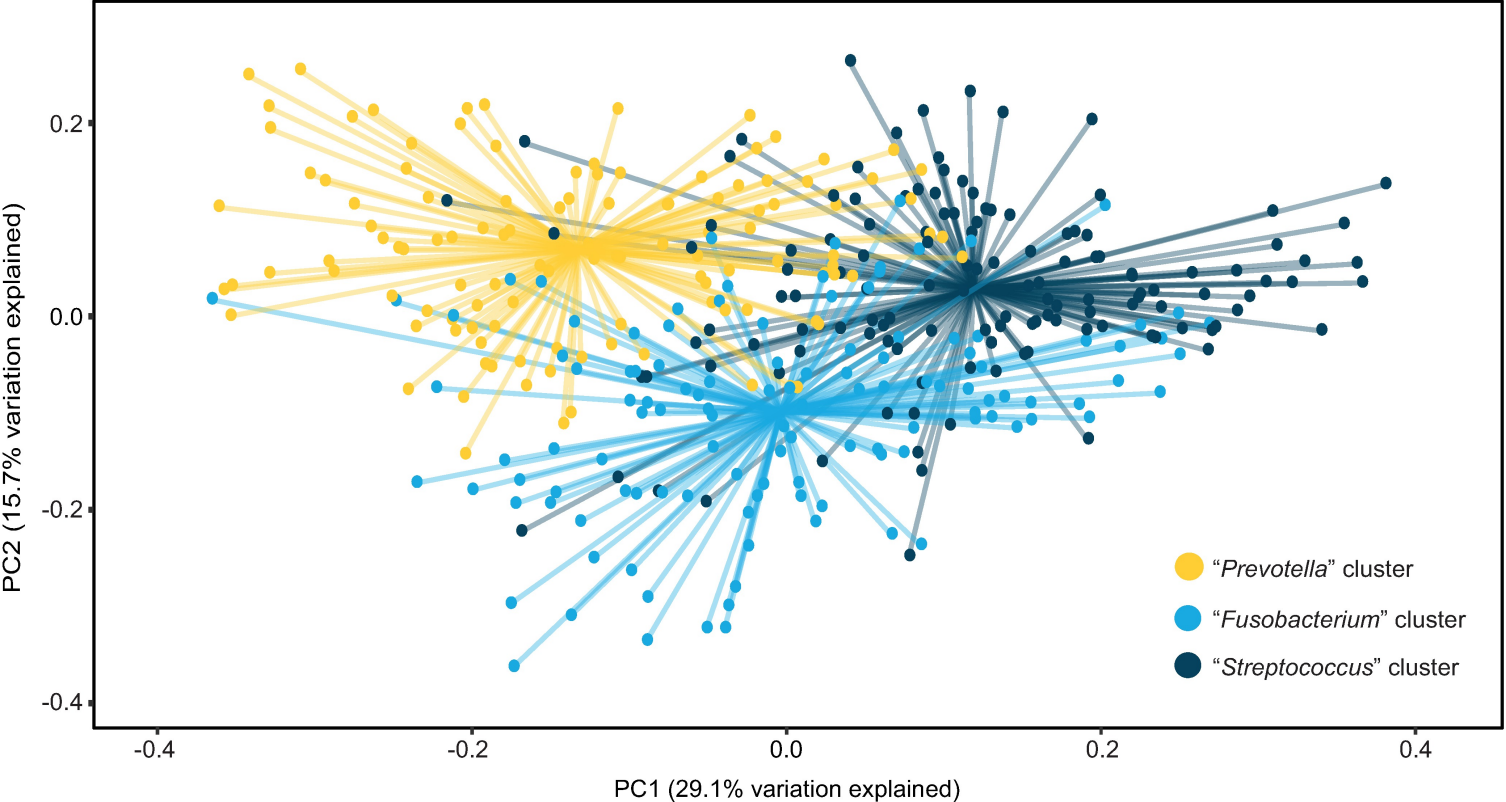
Beta diversity analysis indicates overlapping yet distinct bacterial community clusters as determined by DMM modeling. Bray-Curtis dissimilarity distance varied significantly by DMM cluster (*P*<0.01) and was visualized using principal coordinates analysis. Lines connect samples to cluster centroids. DMM, Dirichlet multinomial mixtures.

**Fig 3:**
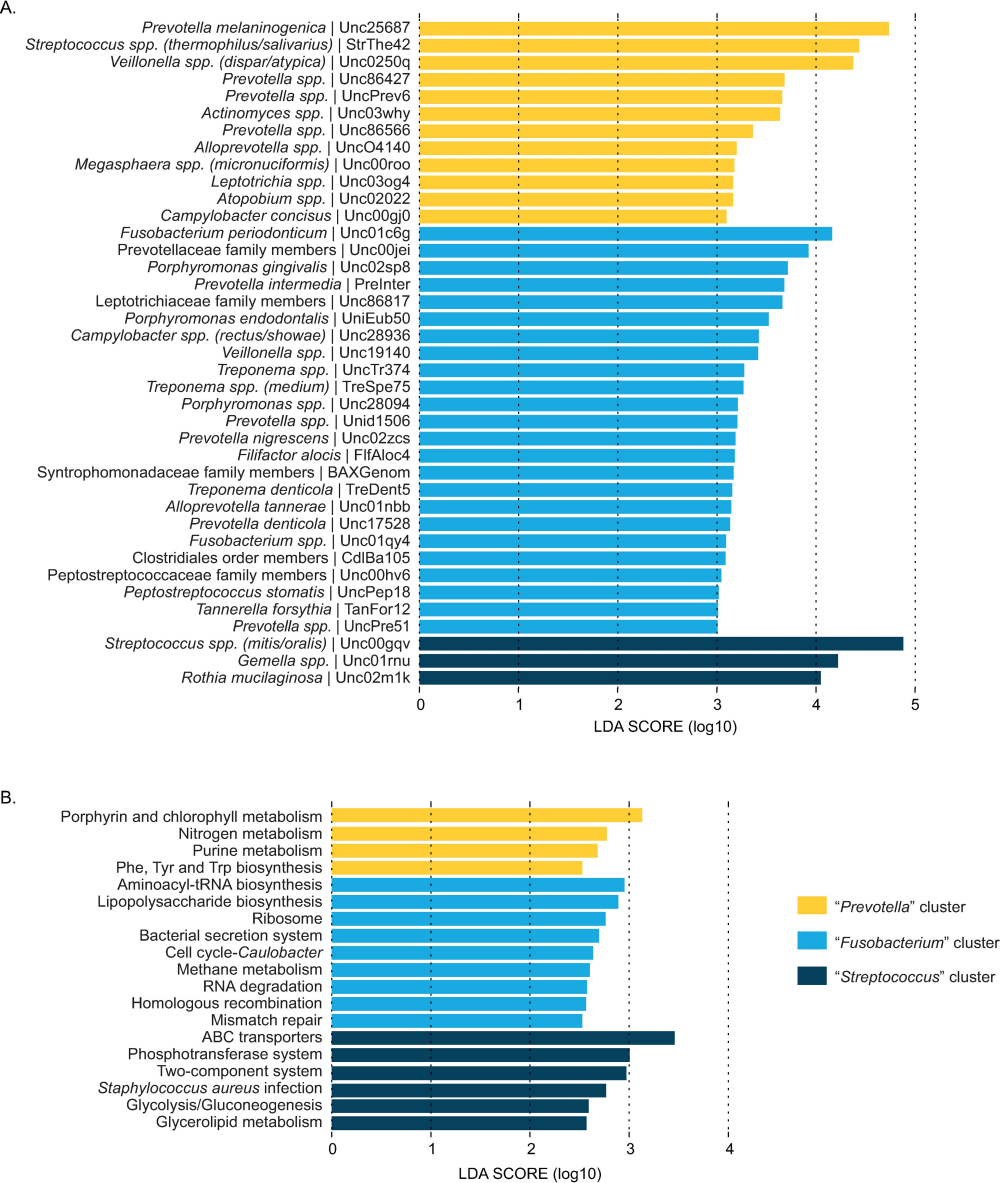
**LEfSe analysis indicates differentially abundant taxa (A) and Tax4Fun-imputed functional pathways (B) by DMM cluster.** Clusters were named for the single OTU with the greatest effect size. LEfSe analyses were conducted using α = 1E-05 and minimum LDA score = 2.5. Due to space limitations, only taxa with a minimum LDA score of 3.0 are shown. A complete list of taxa with minimum LDA score of 2.5 is included in supporting information. DMM, Dirichlet multinomial mixtures; LDA, linear discriminant analysis; LEfSe, LDA effect size.

SparCC bacterial correlation analysis [42] indicated strong potential for differential bacterial community structure (S2 Fig). Hence, we used DMM modeling [43] to cluster samples on the basis of their oral microbiota into three oral community types. Beta diversity assessment indicated overlapping yet distinct bacterial conent in each community (Fig 2). Communities were named “*Streptococcus*,” “*Fusobacterium*,” or “*Prevotella*” based on the foremost differentially abundant OTU as identified by LEfSe [44] (Fig 3A and S3 Table). Bacterial alpha diversity varied significantly between clusters, with observed OTU and Shannon diversity highest among the “*Fusobacterium*” group followed by the “*Prevotella*” and “*Streptococcus*” clusters (Table 2). Consistent with reports associating high bacterial diversity with poor oral health [48], OTUs representing *Fusobacterium periodonticum*, *Porphyromonas gingivalis*, *Treponema denticola*, and *Tannerella forsythia—*all known oral pathogens [49]—were more abundant among “*Fusobacterium*” women (Fig 3A).

**Table 2:** Demographic characteristics of Mexican American women by DMM cluster; values indicate N (%) unless otherwise indicated.

	*Streptococcu*s Cluster (S)^a^	*Fusobacterium* Cluster (F)^a^	*Prevotella* Cluster (P)^a^	Pairwise *P*		
				S vs F	S vs P	F vs P
N, total	133	120	116			
Age (years)						
20–29	17 (12.8)	22 (18.3)	8 (6.9)	<0.01^d^	0.25	<0.01^d^
30–39	54 (40.6)	51 (42.5)	43 (37.1)			
40–49	26 (19.5)	36 (30.0)	32 (27.6)			
≥50	36 (27.1)	11 (9.2)	33 (28.4)			
Education level						
< High school	65 (48.9)	63 (52.5)	55 (47.4)	0.84	0.36	0.27
High school diploma or equivalent	29 (21.8)	24 (20.0)	34 (29.3)			
> High school	38 (28.6)	32 (26.7)	27 (23.3)			
Marital status						
Married	98 (73.7)	91 (75.8)	90 (77.6)	0.61	0.48	0.84
Not married	35 (26.3)	28 (23.3)	26 (22.4)			
Country of birth						
Mexico	98 (73.7)	94 (78.3)	102 (87.9)	0.39	<0.01^d^	0.05
US	35 (26.3)	26 (21.7)	14 (12.1)			
Country of longest residence						
Mexico	64 (48.1)	73 (60.8)	79 (68.1)	0.04	<0.01^d^	0.24
US	69 (51.9)	47 (39.2)	37 (31.9)			
Age of immigration (years)^b^						
0–18	34 (33.3)	21 (22.1)	16 (15.5)	0.13	0.01^d^	0.66
19–24	33 (32.3)	28 (29.5)	34 (29.3)			
25–29	19 (18.6)	20 (21.1)	21 (33.0)			
≥30	16 (15.7)	26 (27.4)	32 (31.1)			
Time in US (years)^b^						
<5	3 (2.9)	14 (14.7)	6 (5.8)	<0.01^d^	0.19	0.12
5–9	14 (13.7)	18 (18.9)	16 (15.5)			
10–14	18 (17.6)	24 (25.3)	30 (29.1)			
15–19	27 (26.5)	22 (23.2)	21 (20.4)			
20–24	14 (13.7)	7 (57.4)	7 (6.8)			
≥25	26 (25.5)	10 (10.5)	23 (22.3)			
English acculturation score						
1–1.75	48 (36.1)	53 (44.2)	51 (44.0)	0.20	<0.01^d^	0.10
2–2.75	32 (24.1)	33 (27.5)	44 (37.9)			
3–4	51 (38.3)	34 (28.3)	21 (18.1)			
Food acculturation^c^						
Only Mexican foods	15 (12.4)	24 (22.2)	16 (15.4)	0.13	0.11	0.13
Mostly Mexican foods	39 (32.2)	33 (30.6)	45 (43.3)			
Mix /Mostly American/Other	67 (55.4)	51 (47.2)	43 (41.3)			
History of alcohol consumption						
No	109 (82.0)	100 (83.3)	101 (87.1)	0.77	0.27	0.42
Yes	24 (18.0)	20 (16.7)	15 (12.9)			
History of farm work						
No	104 (78.2)	101 (84.2)	79 (68.1)	0.23	0.07	<0.01^d^
Yes	29 (21.8)	19 (15.8)	37 (31.9)			
BMI (kg/m^2^)						
Lean (<25)	22 (16.5)	22 (18.3)	19 (16.4)	0.31	0.60	0.94
Overweight (25–29)	50 (37.6)	41 (34.2)	41 (35.3)			
Obese class I (30–34)	42 (31.6)	30 (25.0)	32 (27.6)			
Obese class II+ (≥35)	19 (14.3)	27 (22.5)	24 (20.7)			
Physical activity level (tertiles)						
Light	48 (36.1)	42 (35.0)	42 (36.2)	0.99	0.21	0.28
Moderate	49 (36.8)	44 (36.7)	52 (44.8)			
Heavy	36 (27.1)	31 (25.8)	21 (18.1)			
Shannon diversity, Mean (SD)	2.14 (0.33)	283 (0.38)	2.59 (0.28)	<0.01^d^	<0.01^d^	<0.01^d^
Observed OTUs, Mean (SD)	73.9 (15.4)	125.5 (17.0)	93.8 (18.0)	<0.01^d^	<0.01^d^	<0.01^d^

Abbreviations: BMI, body mass index; DMM, Dirichlet multinomial mixtures; SD, standard deviation.

^a^ Totals may not add up to 100% due to missing responses in <3% of participants.

^b^ Those not born & raised in US; N = 300.

^c^ Variable not assessed prior to 2006; N = 333 for 2006–2011 enrollment.

^d^ *P*-value significant following Sidak correction for multiple comparisons.

The relative abundance of core genera also varied by DMM cluster (S3 Fig). Specifically, the “*Streptococcus*” cluster exhibited the highest levels of *Streptococcus*, *Haemophilus* and *Gemella;* the “*Fusobacterium*” group contained higher amounts of *Fusobacterium*, *Porphyromonas*, *Alloprevotella*, and *Prevotella;* and “*Prevotella*” women exhibited greater abundance of *Prevotella_7*, *Actinomyces* and *Veillonella*.

Differences in predicted functional pathways were also observed by bacterial cluster (Fig 3B), with greater potential for essential amino acid biosynthesis and nitrogen metabolism among the “*Prevotella*” bacterial community type and carbohydrate metabolism and transport among the “*Streptococcus*” group. The “*Fusobacterium*” cluster indicated putative functional differences in DNA replication and repair as well as bacteria-host interactions.

Comparing demographics and health behaviors by DMM cluster, we found the “*Streptococcus*” cluster to be more acculturated, particularly as compared to the *“Prevotella”* group: *“Streptococcus”* participants reported higher English linguistic acculturation scores, were more likely to have been born in the US, or if born in Mexico, to have immigrated at an earlier age (Table 2). In contrast, the “*Prevotella*” group was more likely to report a history of farm work. Both the “*Streptococcus*” and *“Prevotella”* groups were significantly older than the cluster driven by *Fusobacterium* (mean (SD) of 42.2 (1.0) and 43.9 (1.1) vs 37.8 (1.0) years, respectively; *P*<0.01). None of the other variables tested, including BMI and physical activity, differed by cluster assignment.

## Discussion

We examined the oral microbiome in a group of non-smoking Mexican American women from the Houston, Texas, metropolitan area. To our knowledge, this is the first study to characterize the oral microbiota in a large group of first and second generation Mexican American women. We identified a core microbiome of 18 taxa common to 98% or more of women and three microbial community types. Women in the three microbiome clusters varied by age and a variety of acculturation-related variables, including country of birth, country of longest residence, age at immigration, years lived in the US, and acculturation score. Among first generation immigrants, we further observed that time in the US and younger age at immigration were inversely associated with taxonomic richness. Collectively, these results support the potential mutability of the oral microbiota in response to cultural adaptation associated with immigration. They further provide a baseline profile for future studies to investigate relationships between oral bacteria and prospective disease in at-risk Mexican American women.

The oral microbiome is dominated by a handful of genera—*Streptococcus*, *Prevotella*, and *Haemophilus*, to name a few. We observed these and other well-known oral bacteria in our participants, with relative abundance varying by bacterial community type. Of particular note, the “*Prevotella*” and “*Streptococcus*”-defined clusters differed in the relative abundance of nearly all core taxa identified in our study. These clusters also differed demographically, with “*Prevotella*” individuals more likely to have been born in Mexico, to have resided longest in Mexico, and to have immigrated to the US at a later age compared to the “*Streptococcus*” group. Together, these differences support the potential for a microbial transition associated with immigration and adaptation whereby a “*Prevotella*” community dominates in recent Mexican immigrants but transitions to the “*Streptococcus*” signature over time.

The concept of a microbial transition parallels that of acculturation, a likely contributor to any bacterial shift. Observed differences in English linguistic acculturation score [31,32] support this hypothesis, with the “*Streptococcus*” signature dominating among more acculturated women. Furthermore, recent evidence indicates acculturation occurs more rapidly in younger immigrants [50], which is consistent with the earlier US arrival observed among women in the “*Streptococcus*” cluster. Several studies have reported positive relationships between dental care and use of the English language among Hispanics [51–53]. While dental history was not available for the current study, *Streptococcus* species linked with better oral health, including *S*. *mitis* and *S*. *oralis* [49,54,55], were significantly more abundant in the *“Streptococcus”* community type. By comparison, the *“Prevotella”* cluster exhibited higher levels of *S*. *salivarius* and *Veillonella spp*., both of which have been linked to dental caries [54,56]. These observations suggest differences in oral health and/or oral health determinants (e.g., access to dental care) contribute to the relationships observed here. Frequency of dentist visits was only recently incorporated into the MACS questionnaire but appears low; nonetheless, the relationship between dental visits and the oral microbiome could be explored in future studies. Importantly, our work examines the oral microbiota at just a single time point in women enrolled between 2004–2011. The influence of birth or immigration cohort effects cannot be excluded.

Inter-individual variation of the microbiota has previously been observed by geography and culture. Comparing Germans, native Alaskans, and Africans, Li and colleagues observed differential abundances across many common and highly abundant oral bacteria, including *Prevotella*, *Veillonella*, and *Haemophilus* [29]. Takeshita *et al*. reported similar taxonomic differences in a study comparing the salivary microbiome of South Koreans to the Japanese [28]—two populations with arguably fewer differences in diet and host genetic variation. These studies support the idea of a core human oral microbiome that, despite differences in relative taxa abundance, provides functional consistency and stability across cultures. Data from the Human Microbiome Project support this assertion, with the taxonomic composition of the buccal mucosa exhibiting far more variability than its microbial metabolic pathway content [47]. Hence, while our data suggest acculturation in Mexican American women may be accompanied by compositional changes of the oral microbiota, differences in the overarching function of these communities are likely small. Our own analysis supports this hypothesis as the variation in putative functional pathways between our bacterial community clusters was more modest than their observed taxonomic differences. Consequently, many roads may lead to a “healthy” oral microbiome.

In addition to the “*Prevotella*” and “*Streptococcus*” clusters, we observed a third group of women defined by higher levels of moderate- to high-risk periodontal pathogens. Presence of pathogens was further reflected in putative functional differences, with bacteria-host interaction and cellular turnover/repair pathways dominating the *“Fusobacterium”* cluster. One-third of our participants presented with this bacterial signature, suggesting periodontal disease or risk thereof may be widespread within our cohort. History of periodontitis was not assessed in the MACS study, but prevalence of this inflammatory disease is higher among Hispanics/Latinos according to national surveys [57]. Consistent with these observations, Mason *et al*. recently reported greater abundance of *Porphyromonas*, *Treponema*, and *Fusobacterium spp*. in the subgingival microbiota of Latinos as compared to non-Hispanic whites and non-Hispanic blacks [58]. These disparities may in part be due to chronic inflammation as a result of the physical, psychosocial, and cultural stressors that accompany immigration. Notably, Miranda and Matheny found that acculturative stress was inversely related to time in the US among adult Latinos [59], and women in the *“Fusobacterium”* cluster were more likely to report living in the US less than 10 years.

Interest in the oral microbiome is rising as scientists identify ever more associations between bacteria of the mouth and complex disease. Many of these associations stem from links to periodontitis and associated diseases, which may reflect an underlying susceptibility or avenue toward systemic inflammation. Although the directionality of these relationships is often unclear, large cohorts such as MACS provide the opportunity to elucidate the timing of such relationships and explore the oral microbiome as a potential biomarker or etiologic underpinning of disease. Moreover, our study shows that MACS mouthwash samples, initially collected for human genetic studies and kept in frozen storage for up to 15 years, produce high quality microbial sequencing data; hence, studies to examine the oral microbiome and incident disease are underway. Characterizing the oral microbiome in an effort to identify those at highest disease risk will help us better target health interventions to those with greatest need. As a putative comprehensive assessment of biological, behavioral, and environmental risk factors, the oral microbiome may prove to be one of the most informative and easily accessible biomarkers for research in low income, resource poor populations.

## Conclusions

First and second generation Mexican American women face a number of health issues modulated by acculturation and adaptation to life in the US. The oral microbiota, itself linked to many of these conditions, also appears to differ by factors associated with immigration among Mexican American women and has the potential to impact host health via a multitude of functions. Whether and how differences in oral health or oral health care contribute to these relationships warrant further research and could have broad implications for how we target public health problems in this burgeoning population.

## Supporting information

S1 Fig Bacterial diversity by age and country of longest residence among Mexican American women. Bacterial richness as measured by observed OTU (A) and Chao index (C) is inversely associated with age in women who have lived >50% of their life in the US (observed OTU: r = -0.28, P<0.01; Chao: r = -0.32, P<0.01) versus Mexico (observed OTU: r = -0.07, P = 0.31; Chao: r = -0.05, P = 0.46). Among women who resided longer in the US, country of birth did not affect this relationship (US vs Mexico, P = 0.56 for observed OTU and P = 0.87 for Chao) (B & D). (E) Shannon diversity did not vary with age, irrespective of country of longest residence (US, P = 0.14; Mexico, P = 0.12). Among women residing longer in the US, the relationship between Shannon diversity and age was not modified by country of birth (US vs Mexico, P = 0.22) (F). OTU, operational taxonomic unit.
(PDF)

S2 Fig OTU co-occurrence relationships identified by SparCC correlation analysis among Mexican American women. OTU co-occurrence relationships among Mexican American women identified by SparCC correlation analysis. Analysis was restricted to OTUs detected at ≥0.1% relative abundance in at least one-third of samples. Positive correlations (co-occurrence) are shown in shades of blue and negative correlations (co-exclusion) in red. Correlation strength is indicated by circle size, with larger circles depicting stronger associations. Only significant relationships are shown (FDR Q = 0.1). Black rectangles demarcate results of hierarchical clustering.
(PDF)

S3 Fig Relative abundance of “core” genera in Mexican American women by DMM cluster. Relative abundance of “core” genera in Mexican American women by DMM cluster. (A) Stacked bar graphs indicate the proportion of each sample represented by each core genus. (B) Box-plots of core taxa by DMM cluster. Post-multiple comparison adjustment, pairwise P<0.05: ^a^ “*Streptococcus*” cluster vs “*Fusobacterium*” cluster; ^b^ “*Streptococcus*” cluster vs “*Prevotella*” cluster; ^c^ “*Fusobacterium*” cluster vs “*Prevotella*” cluster.
(PDF)

S1 Table Chao diversity index by demographic characteristics in Mexican American women (N = 369). (PDF)

S2 Table Shannon diversity index by demographic characteristics in Mexican American women (N = 369). (PDF)

S3 Table Differential OTU-level taxa by DMM cluster as determined by LEfSe. (PDF)

S1 File Participant demographic data. (XLSX)

S2 File Rarefied OTU table. (XLSX)

## References

[1] U.S. Census Bureau, Population Division. Annual estimates of the resident population by sex, age, race, and Hispanic origin for the United States and states: April 1, 2010 to July 1, 2015; 2016. [cited 2016 Sep 9]. Database: American FactFinder [Internet]. Available from: http://www.factfinder.census.gov.

[2] Generational differences. Report No. 7054. Pew Hispanic Center. 2004. Available from: http://www.pewhispanic.org/files/2011/10/13.pdf.

[3] U.S. Census Bureau. American Community Survey 1-year estimates; 2016. [cited 2016 Sep 9]. Database: American FactFinder [Internet]. Available from: http://www.factfinder.census.gov.

[4] National Center for Health Statistics. Health, United States, 2015: with special feature on racial and ethnic health disparities. Report No. 2016–1232. Hyattsville, MD: US Department of Health and Human Services; 2016. 27308685

[5] Siegel RL, Miller KD, Jemal A. Cancer statistics, 2016. CA Cancer J Clin. 2016; 66: 7–30. doi: 10.3322/caac.21332 26742998 10.3322/caac.21332

[6] American Cancer Society. Cancer facts & figures for Hispanics/Latinos 2015–2017. Report No. 862315. Atlanta, GA: American Cancer Society; 2015.

[7] Albrecht SS, Diez Roux AV, Aiello AE, Schulz AJ, Abraido-Lanza AF. Secular trends in the association between nativity/length of US residence with body mass index and waist circumference among Mexican-Americans, 1988–2008. Int J Public Health. 2013; 58: 573–581. doi: 10.1007/s00038-012-0414-5 23052250 10.1007/s00038-012-0414-5 PMC3570586

[8] Garcia L, Gold EB, Wang L, Yang X, Mao M, Schwartz AV. The relation of acculturation to overweight, obesity, pre-diabetes and diabetes among U.S. Mexican-American women and men. Ethn Dis. 2012; 22: 58–64. 22774310 PMC4203316

[9] Barcenas CH, Wilkinson AV, Strom SS, Cao Y, Saunders KC, Mahabir S, et al Birthplace, years of residence in the United States, and obesity among Mexican-American adults. Obesity (Silver Spring). 2007; 15: 1043–1052. 17426341 10.1038/oby.2007.537

[10] O'Brien MJ, Alos VA, Davey A, Bueno A, Whitaker RC. Acculturation and the prevalence of diabetes in US Latino adults, National Health and Nutrition Examination Survey 2007–2010. Prev Chronic Dis. 2014; 11: E176 doi: 10.5888/pcd11.140142 25299982 10.5888/pcd11.140142 PMC4193061

[11] Anderson C, Zhao H, Daniel CR, Hromi-Fiedler A, Dong Q, Elhor Gbito KY, et al Acculturation and diabetes risk in the Mexican American Mano a Mano Cohort. Am J Public Health. 2016; 106: 547–549. doi: 10.2105/AJPH.2015.303008 26794174 10.2105/AJPH.2015.303008 PMC4815959

[12] Daviglus ML, Talavera GA, Aviles-Santa ML, Allison M, Cai J, Criqui MH, et al Prevalence of major cardiovascular risk factors and cardiovascular diseases among Hispanic/Latino individuals of diverse backgrounds in the United States. JAMA. 2012; 308: 1775–1784. doi: 10.1001/jama.2012.14517 23117778 10.1001/jama.2012.14517 PMC3777250

[13] Daviglus ML, Pirzada A, Durazo-Arvizu R, Chen J, Allison M, Aviles-Santa L, et al Prevalence of low cardiovascular risk profile among diverse Hispanic/Latino adults in the United States by age, sex, and level of acculturation: The Hispanic Community Health Study/Study of Latinos. J Am Heart Assoc. 2016; 5: e003929 doi: 10.1161/JAHA.116.003929 27543802 10.1161/JAHA.116.003929 PMC5015308

[14] Humphrey LL, Fu R, Buckley DI, Freeman M, Helfand M. Periodontal disease and coronary heart disease incidence: a systematic review and meta-analysis. J Gen Intern Med. 2008; 23: 2079–2086. doi: 10.1007/s11606-008-0787-6 18807098 10.1007/s11606-008-0787-6 PMC2596495

[15] Janket SJ, Baird AE, Chuang SK, Jones JA. Meta-analysis of periodontal disease and risk of coronary heart disease and stroke. Oral Surg Oral Med Oral Pathol Oral Radiol Endod. 2003; 95: 559–569. doi: 10.1067/moe.2003.107 12738947 10.1067/moe.2003.107

[16] Mercado F, Marshall RI, Klestov AC, Bartold PM. Is there a relationship between rheumatoid arthritis and periodontal disease? J Clin Periodontol. 2000; 27: 267–272. 10783841 10.1034/j.1600-051x.2000.027004267.x

[17] Michaud DS, Liu Y, Meyer M, Giovannucci E, Joshipura K. Periodontal disease, tooth loss, and cancer risk in male health professionals: a prospective cohort study. Lancet Oncol. 2008; 9: 550–558. doi: 10.1016/S1470-2045(08)70106-2 18462995 10.1016/S1470-2045(08)70106-2 PMC2601530

[18] Bierne H, Hamon M, Cossart P. Epigenetics and bacterial infections. Cold Spring Harb Perspect Med. 2012; 2: a010272 doi: 10.1101/cshperspect.a010272 23209181 10.1101/cshperspect.a010272 PMC3543073

[19] Belstrom D, Holmstrup P, Bardow A, Kokaras A, Fiehn NE, Paster BJ. Temporal stability of the salivary microbiota in oral health. PLoS One. 2016; 11: e0147472 doi: 10.1371/journal.pone.0147472 26799067 10.1371/journal.pone.0147472 PMC4723053

[20] Zaura E, Brandt BW, Teixeira de Mattos MJ, Buijs MJ, Caspers MP, Rashid MU, et al Same exposure but two radically different responses to antibiotics: resilience of the salivary microbiome versus long-term microbial shifts in feces. MBio. 2015; 6: e01693–15. doi: 10.1128/mBio.01693-15 26556275 10.1128/mBio.01693-15 PMC4659469

[21] David LA, Materna AC, Friedman J, Campos-Baptista MI, Blackburn MC, Perrotta A, et al Host lifestyle affects human microbiota on daily timescales. Genome Biol. 2014; 15: R89 doi: 10.1186/gb-2014-15-7-r89 25146375 10.1186/gb-2014-15-7-r89 PMC4405912

[22] Wu J, Peters BA, Dominianni C, Zhang Y, Pei Z, Yang L, et al Cigarette smoking and the oral microbiome in a large study of American adults. ISME J. 2016; 10:2435–2446. doi: 10.1038/ismej.2016.37 27015003 10.1038/ismej.2016.37 PMC5030690

[23] Kato I, Vasquez A, Moyerbrailean G, Land S, Djuric Z, Sun J, et al Nutritional correlates of human oral microbiome. J Am Coll Nutr. 2017; 36: 88–98. doi: 10.1080/07315724.2016.1185386 27797671 10.1080/07315724.2016.1185386 PMC5477991

[24] Corby PM, Biesbrock A, Bartizek R, Corby AL, Monteverde R, Ceschin R, et al Treatment outcomes of dental flossing in twins: molecular analysis of the interproximal microflora. J Periodontol. 2008; 79: 1426–1433. doi: 10.1902/jop.2008.070585 18672992 10.1902/jop.2008.070585

[25] Huang S, Yang F, Zeng X, Chen J, Li R, Wen T, et al Preliminary characterization of the oral microbiota of Chinese adults with and without gingivitis. BMC Oral Health. 2011; 11: 33 doi: 10.1186/1472-6831-11-33 22152152 10.1186/1472-6831-11-33 PMC3254127

[26] Yang F, Zeng X, Ning K, Liu KL, Lo CC, Wang W, et al Saliva microbiomes distinguish caries-active from healthy human populations. ISME J. 2012; 6: 1–10. doi: 10.1038/ismej.2011.71 21716312 10.1038/ismej.2011.71 PMC3246229

[27] Nasidze I, Li J, Quinque D, Tang K, Stoneking M. Global diversity in the human salivary microbiome. Genome Res. 2009; 19: 636–643. doi: 10.1101/gr.084616.108 19251737 10.1101/gr.084616.108 PMC2665782

[28] Takeshita T, Matsuo K, Furuta M, Shibata Y, Fukami K, Shimazaki Y, et al Distinct composition of the oral indigenous microbiota in South Korean and Japanese adults. Sci Rep. 2014; 4: 6990 doi: 10.1038/srep06990 25384884 10.1038/srep06990 PMC4227031

[29] Li J, Quinque D, Horz HP, Li M, Rzhetskaya M, Raff JA, et al Comparative analysis of the human saliva microbiome from different climate zones: Alaska, Germany, and Africa. BMC Microbiol. 2014; 14: 316 doi: 10.1186/s12866-014-0316-1 25515234 10.1186/s12866-014-0316-1 PMC4272767

[30] Chow WH, Chrisman M, R Daniel C, Ye Y, Gomez H, Dong Q, et al Cohort Profile: The Mexican American Mano a Mano Cohort. Int J Epidemiol; Forthcoming. doi: 10.1093/ije/dyv016 25747868 10.1093/ije/dyv016 PMC6251595

[31] Marin G. A new measurement of acculturation for Hispanics: the bidimensional acculturation scale for Hispanics (BAS). Hisp J Behav Sci. 1996; 18: 297–316.

[32] Mendoza R. An empirical scale to measure type and degree of acculturation in Mexican-American adolescents and adults. J Cross-Cultural Psyschol. 1989; 20: 375–385.

[33] Mai PL, Sullivan-Halley J, Ursin G, Stram DO, Deapen D, Villaluna D, et al Physical activity and colon cancer risk among women in the California Teachers Study. Cancer Epidemiol Biomarkers Prev. 2007; 16: 517–525. doi: 10.1158/1055-9965.EPI-06-0747 17372247 10.1158/1055-9965.EPI-06-0747

[34] Caporaso JG, Lauber CL, Walters WA, Berg-Lyons D, Lozupone CA, Turnbaugh PJ, et al Global patterns of 16S rRNA diversity at a depth of millions of sequences per sample. Proc Natl Acad Sci U S A. 2011; 108 Suppl 1: 4516–4522. 20534432 10.1073/pnas.1000080107 PMC3063599

[35] Edgar RC. Search and clustering orders of magnitude faster than BLAST. Bioinformatics. 2010; 26: 2460–2461. doi: 10.1093/bioinformatics/btq461 20709691 10.1093/bioinformatics/btq461

[36] Langmead B, Salzberg SL. Fast gapped-read alignment with Bowtie 2. Nat Methods. 2012; 9: 357–359. doi: 10.1038/nmeth.1923 22388286 10.1038/nmeth.1923 PMC3322381

[37] Edgar RC. UPARSE: highly accurate OTU sequences from microbial amplicon reads. Nat Methods. 2013; 10: 996–998. doi: 10.1038/nmeth.2604 23955772 10.1038/nmeth.2604

[38] Zhang Z, Schwartz S, Wagner L, Miller W. A greedy algorithm for aligning DNA sequences. J Comput Biol. 2000; 7: 203–214. doi: 10.1089/10665270050081478 10890397 10.1089/10665270050081478

[39] Asshauer KP, Wemheuer B, Daniel R, Meinicke P. Tax4Fun: predicting functional profiles from metagenomic 16S rRNA data. Bioinformatics. 2015; 31: 2882–2884. doi: 10.1093/bioinformatics/btv287 25957349 10.1093/bioinformatics/btv287 PMC4547618

[40] Kanehisa M, Goto S. KEGG: Kyoto encyclopedia of genes and genomes. Nucleic Acids Res. 2000; 28: 27–30. 10592173 10.1093/nar/28.1.27 PMC102409

[41] Kanehisa M, Sato Y, Kawashima M, Furumichi M, Tanabe M. KEGG as a reference resource for gene and protein annotation. Nucleic Acids Res. 2016; 44: D457–62. doi: 10.1093/nar/gkv1070 26476454 10.1093/nar/gkv1070 PMC4702792

[42] Friedman J, Alm EJ. Inferring correlation networks from genomic survey data. PLoS Comput Biol. 2012; 8: e1002687 doi: 10.1371/journal.pcbi.1002687 23028285 10.1371/journal.pcbi.1002687 PMC3447976

[43] Holmes I, Harris K, Quince C. Dirichlet multinomial mixtures: generative models for microbial metagenomics. PLoS One. 2012; 7: e30126 doi: 10.1371/journal.pone.0030126 22319561 10.1371/journal.pone.0030126 PMC3272020

[44] Segata N, Izard J, Waldron L, Gevers D, Miropolsky L, Garrett WS, et al Metagenomic biomarker discovery and explanation. Genome Biol. 2011; 12: R60 doi: 10.1186/gb-2011-12-6-r60 21702898 10.1186/gb-2011-12-6-r60 PMC3218848

[45] Alkek Center for Metagenomics and Microbiome Research, Baylor College of Medicine. Sage microbiome explorer. 2015. Available from: http://sage.mx.

[46] R Core Team, R Foundation for Statistical Computing. R: a language and environment for statistical computing. 2016. Available from: http://www.r-project.org.

[47] Human Microbiome Project Consortium. Structure, function and diversity of the healthy human microbiome. Nature. 2012; 486: 207–214. doi: 10.1038/nature11234 22699609 10.1038/nature11234 PMC3564958

[48] Griffen AL, Beall CJ, Campbell JH, Firestone ND, Kumar PS, Yang ZK, et al Distinct and complex bacterial profiles in human periodontitis and health revealed by 16S pyrosequencing. ISME J. 2012; 6: 1176–1185. doi: 10.1038/ismej.2011.191 22170420 10.1038/ismej.2011.191 PMC3358035

[49] Socransky SS, Haffajee AD, Cugini MA, Smith C, Kent RL Jr. Microbial complexes in subgingival plaque. J Clin Periodontol. 1998; 25: 134–144. 9495612 10.1111/j.1600-051x.1998.tb02419.x

[50] Cheung BY, Chudek M, Heine SJ. Evidence for a sensitive period for acculturation: younger immigrants report acculturating at a faster rate. Psychol Sci. 2011; 22: 147–152. doi: 10.1177/0956797610394661 21189354 10.1177/0956797610394661

[51] Graham MA, Tomar SL, Logan HL. Perceived social status, language and identified dental home among Hispanics in Florida. J Am Dent Assoc. 2005; 136: 1572–1582. 16329424 10.14219/jada.archive.2005.0092

[52] Riley JL 3rd, Gibson E, Zsembik BA, Duncan RP, Gilbert GH, Heft MW. Acculturation and orofacial pain among Hispanic adults. J Pain. 2008; 9: 750–758. doi: 10.1016/j.jpain.2008.03.007 18456564 10.1016/j.jpain.2008.03.007 PMC3597078

[53] Flores G, Tomany-Korman SC. The language spoken at home and disparities in medical and dental health, access to care, and use of services in US children. Pediatrics. 2008; 121: e1703–14. doi: 10.1542/peds.2007-2906 18519474 10.1542/peds.2007-2906

[54] Gross EL, Beall CJ, Kutsch SR, Firestone ND, Leys EJ, Griffen AL. Beyond *Streptococcus mutans*: dental caries onset linked to multiple species by 16S rRNA community analysis. PLoS One. 2012; 7: e47722 doi: 10.1371/journal.pone.0047722 23091642 10.1371/journal.pone.0047722 PMC3472979

[55] Corby PM, Lyons-Weiler J, Bretz WA, Hart TC, Aas JA, Boumenna T, et al Microbial risk indicators of early childhood caries. J Clin Microbiol. 2005; 43: 5753–5759. doi: 10.1128/JCM.43.11.5753-5759.2005 16272513 10.1128/JCM.43.11.5753-5759.2005 PMC1287835

[56] Kanasi E, Dewhirst FE, Chalmers NI, Kent R Jr, Moore A, Hughes CV, et al Clonal analysis of the microbiota of severe early childhood caries. Caries Res. 2010; 44: 485–497. doi: 10.1159/000320158 20861633 10.1159/000320158 PMC2975730

[57] Eke PI, Dye BA, Wei L, Slade GD, Thornton-Evans GO, Borgnakke WS, et al Update on prevalence of periodontitis in adults in the United States: NHANES 2009 to 2012. J Periodontol. 2015; 86: 611–622. doi: 10.1902/jop.2015.140520 25688694 10.1902/jop.2015.140520 PMC4460825

[58] Mason MR, Nagaraja HN, Camerlengo T, Joshi V, Kumar PS. Deep sequencing identifies ethnicity-specific bacterial signatures in the oral microbiome. PLoS One. 2013; 8: e77287 doi: 10.1371/journal.pone.0077287 24194878 10.1371/journal.pone.0077287 PMC3806732

[59] Miranda AO and Matheny KB. Socio-psychological predictors of acculturative stress among Latino adults. J Ment Health Couns. 2000; 22: 306–317.

