# Supplementary figures and images for: Oral microbiota reveals signs of acculturation in Mexican American women
Source: PLoS One. 2018 Apr 25;13(4):e0194100. doi: 10.1371/journal.pone.0194100 (PMC5918619; doi:10.1371/journal.pone.0194100)

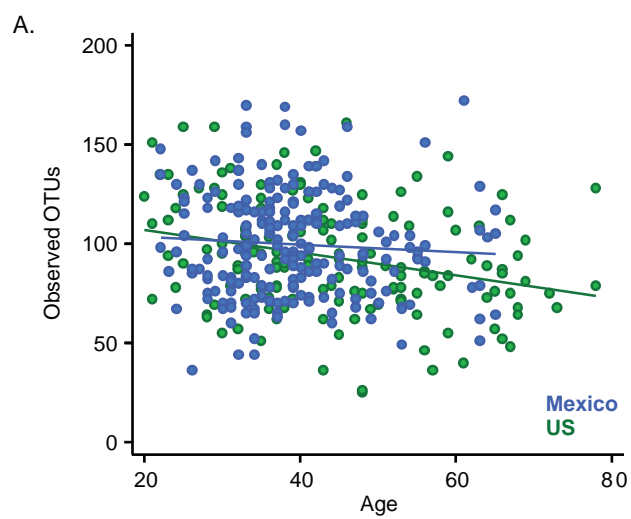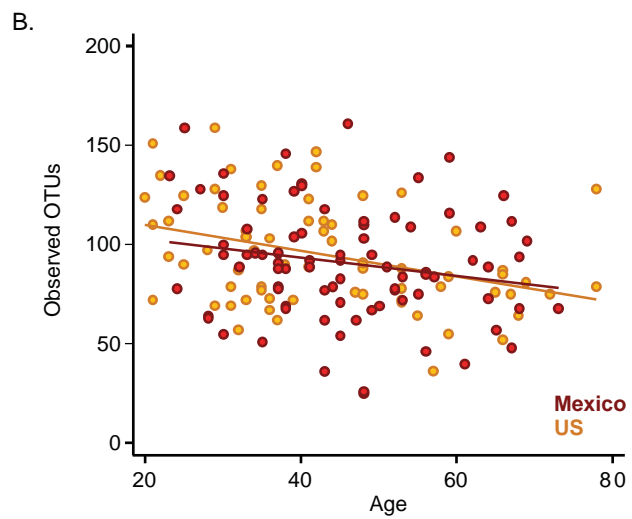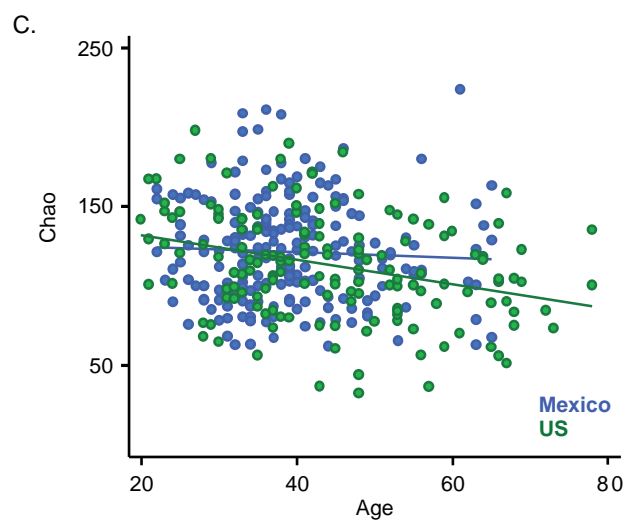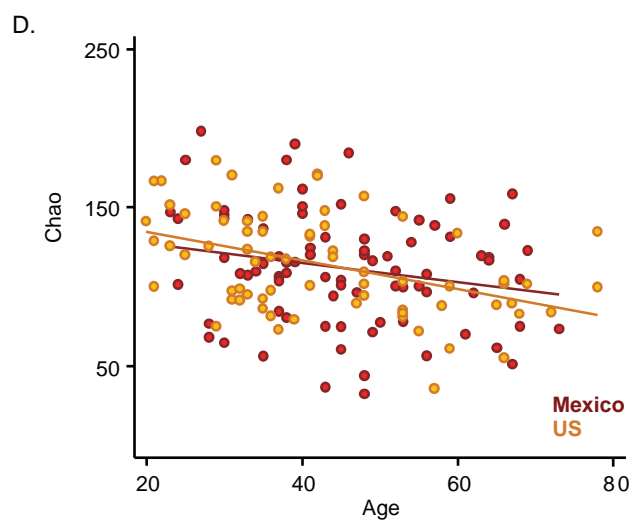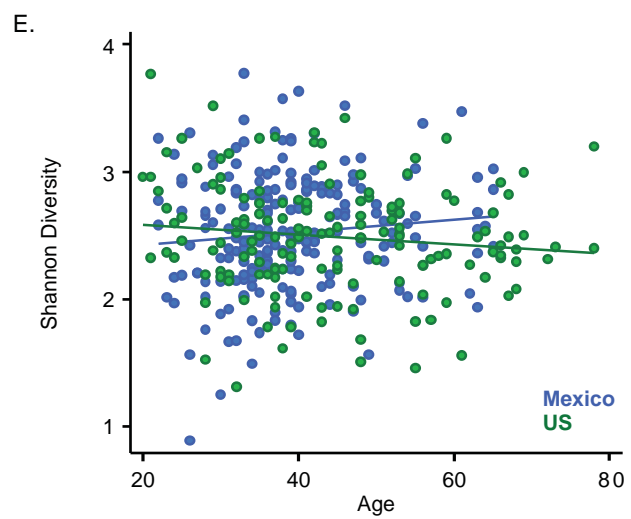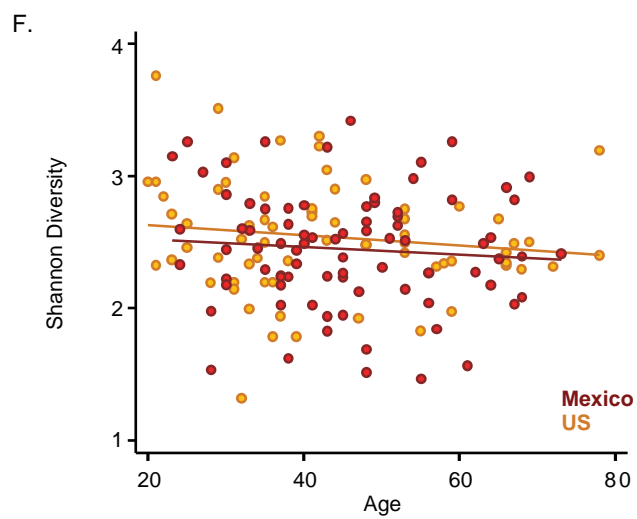

S1 Fig

Supplement: S1 Fig — Bacterial richness as measured by observed OTU (A) and Chao index (C) is inversely associated with age in women who have lived >50% of their life in the US (observed OTU: r = -0.28, P<0.01; Chao: r = -0.32, P<0.01) versus Mexico (observed OTU: r = -0.07, P = 0.31; Chao: r = -0.05, P = 0.46). Among women who resided longer in the US, country of birth did not affect this relationship (US vs Mexico, P = 0.56 for observed OTU and P = 0.87 for Chao) (B & D). (E) Shannon diversity did not vary with age, irrespective of country of longest residence (US, P = 0.14; Mexico, P = 0.12). Among women residing longer in the US, the relationship between Shannon diversity and age was not modified by country of birth (US vs Mexico, P = 0.22) (F). OTU, operational taxonomic unit. (PDF) [file pone.0194100.s001.pdf]

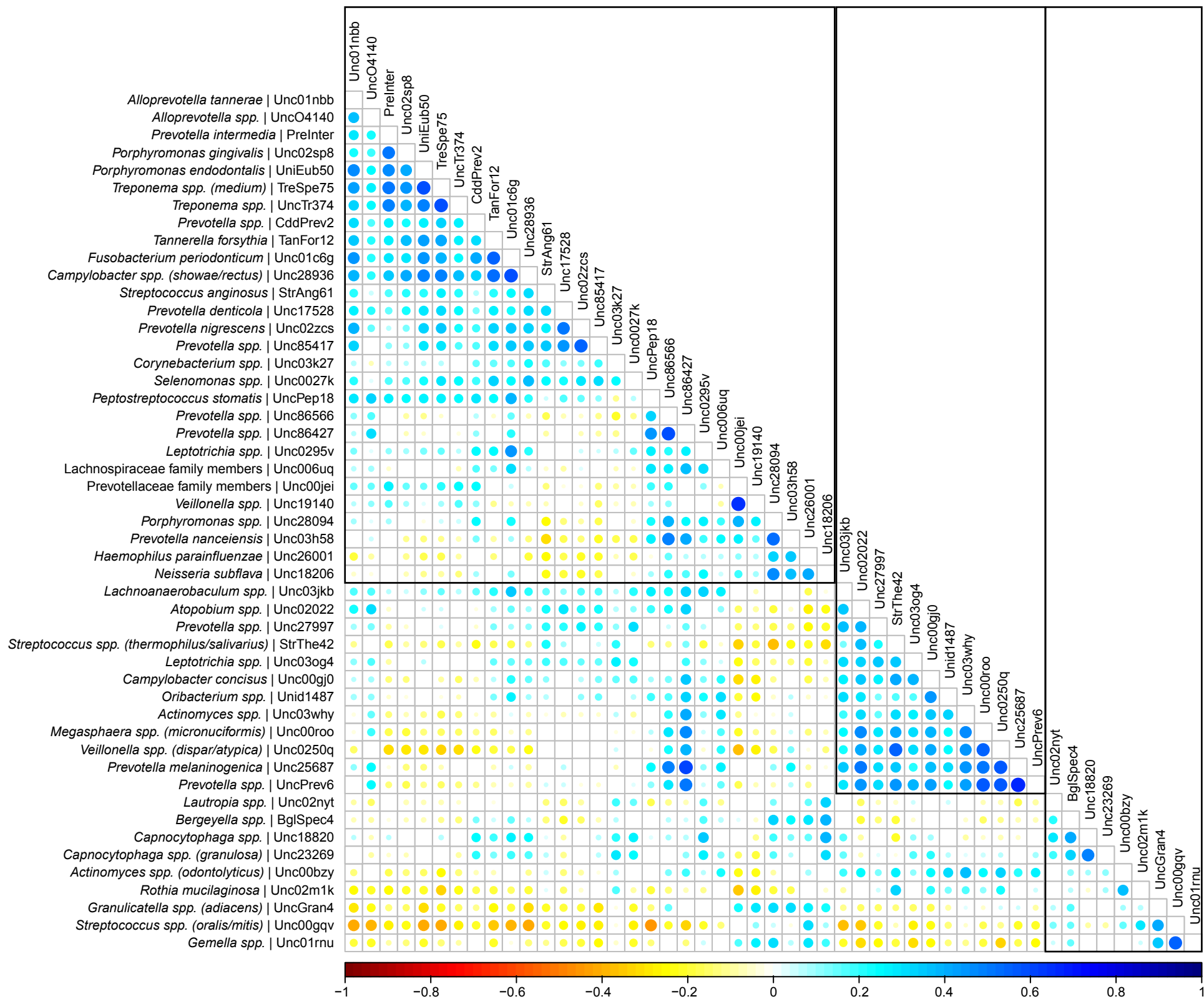

S2 Fig

Supplement: S2 Fig — OTU co-occurrence relationships among Mexican American women identified by SparCC correlation analysis. Analysis was restricted to OTUs detected at ≥0.1% relative abundance in at least one-third of samples. Positive correlations (co-occurrence) are shown in shades of blue and negative correlations (co-exclusion) in red. Correlation strength is indicated by circle size, with larger circles depicting stronger associations. Only significant relationships are shown (FDR Q = 0.1). Black rectangles demarcate results of hierarchical clustering. (PDF) [file pone.0194100.s002.pdf]

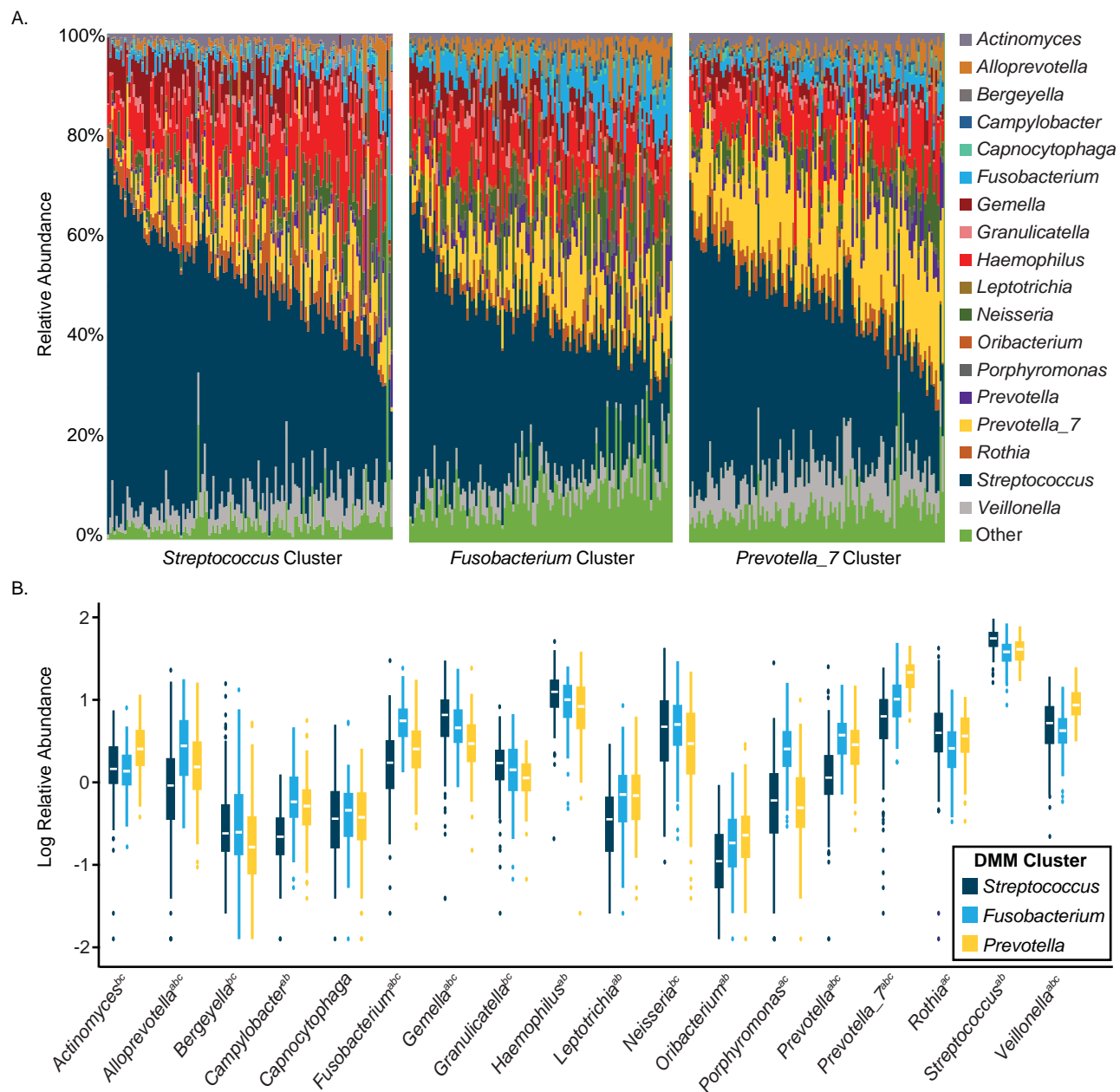

S3 Fig

Supplement: S3 Fig — Relative abundance of “core” genera in Mexican American women by DMM cluster. (A) Stacked bar graphs indicate the proportion of each sample represented by each core genus. (B) Box-plots of core taxa by DMM cluster. Post-multiple comparison adjustment, pairwise P<0.05: a “Streptococcus” cluster vs “Fusobacterium” cluster; b “Streptococcus” cluster vs “Prevotella” cluster; c “Fusobacterium” cluster vs “Prevotella” cluster. (PDF) [file pone.0194100.s003.pdf]
