# Supplementary material for: Oral microbiota reveals signs of acculturation in Mexican American women
Source: PLoS One. 2018 Apr 25;13(4):e0194100. doi: 10.1371/journal.pone.0194100 (PMC5918619; doi:10.1371/journal.pone.0194100)
Supplement: S2 Table — (PDF) [file pone.0194100.s005.pdf]

S2 Table. Shannon diversity index by demographic characteristics in Mexican American women (N=369 unless otherwise indicated).

		Unadjusted		Adjusted for age		Adjusted for age & education	
	N (%)[a]	Mean (SE)	*P*	Mean (SE)	*P*	Mean (SE)	*P*
Age (years)							
20-29	47 (12.7)	2.57 (0.06)	0.30				
30-39	148 (40.1)	2.47 (0.04)					
40-49	94 (25.5)	2.56 (0.05)					
≥50	80 (21.7)	2.49 (0.05)					
Education level							
< High school	183 (49.6)	2.51 (0.03)	0.72	2.51 (0.03)	0.72		
High school diploma or equivalent	87 (23.6)	2.48 (0.05)		2.48 (0.05)			
> High school	97 (26.3)	2.54 (0.04)		2.53 (0.04)			
Marital status							
Married	279 (75.6)	2.52 (0.03)	0.35	2.52 (0.03)	0.36	2.52 (0.03)	0.30
Not married	89 (24.1)	2.47 (0.05)		2.47 (0.05)		2.46 (0.05)	
Country of birth							
Mexico	294 (79.7)	2.51 (0.03)	0.86	2.51 (0.03)	0.86	2.51 (0.03)	0.89
US	75 (20.3)	2.52 (0.05)		2.52 (0.05)		2.52 (0.05)	
Country of longest residence							
Mexico	216 (58.5)	2.52 (0.03)	0.49	2.52 (0.03)	0.52	2.52 (0.03)	0.59
US	153 (41.5)	2.49 (0.04)		2.49 (0.04)		2.49 (0.04)	
Age of immigration (years)[b]							
0-18	71 (23.7)	2.38 (0.05)	0.07	2.38 (0.05)	0.06	2.39 (0.06)	0.10
19-24	95 (31.7)	2.51 (0.05)		2.51 (0.05)		2.51 (0.05)	
25-29	60 (20.0)	2.58 (0.06)		2.59 (0.06)		2.59 (0.06)	
≥30	74 (24.7)	2.53 (0.05)		2.54 (0.06)		2.54 (0.06)	
Time in US (years)[b]							
<5	23 (7.7)	2.58 (0.09)	0.53	2.61 (0.10)	0.33	2.60 (0.10)	0.42
5-9	48 (16.0)	2.47 (0.06)		2.49 (0.07)		2.49 (0.07)	
10-14	72 (24.0)	2.57 (0.05)		2.58 (0.05)		2.58 (0.06)	
15-19	70 (23.3)	2.48 (0.05)		2.49 (0.05)		2.50 (0.06)	
20-24	28 (9.3)	2.50 (0.08)		2.48 (0.09)		2.48 (0.09)	
≥25	59 (19.7)	2.43 (0.06)		2.39 (0.07)		2.40 (0.07)	
English acculturation score							
1-1.75	152 (41.2)	2.53 (0.04)	0.37	2.53 (0.04)	0.37	2.54 (0.04)	0.28
2-2.75	109 (29.5)	2.54 (0.04)		2.54 (0.04)		2.54 (0.04)	
3-4	106 (28.7)	2.46 (0.04)		2.46 (0.04)		2.46 (0.04)	
Food acculturation[c]							
Only Mexican foods	55 (16.5)	2.48 (0.06)	0.57	2.48 (0.06)	0.58	2.47 (0.06)	0.55
Mostly Mexican foods	117 (35.1)	2.51 (0.04)		2.51 (0.04)		2.52 (0.04)	
Mix /Mostly American/Other	161 (48.3)	2.46 (0.03)		2.46 (0.03)		2.46 (0.03)	
History of alcohol consumption							
No	310 (84.0)	2.51 (0.03)	0.55	2.51 (0.03)	0.55	2.52 (0.03)	0.57
Yes	59 (16.0)	2.48 (0.06)		2.48 (0.06)		2.48 (0.06)	
History of farm work							
No	284 (77.0)	2.52 (0.03)	0.49	2.52 (0.03)	0.50	2.52 (0.03)	0.55
Yes	85 (23.0)	2.48 (0.05)		2.48 (0.05)		2.48 (0.05)	
BMI (kg/m^2)							
Lean (<25)	63 (17.1)	2.54 (0.06)	0.70	2.54 (0.06)	0.70	2.54 (0.06)	0.76
Overweight (25-29)	132 (35.8)	2.51 (0.04)		2.51 (0.04)		2.51 (0.04)	
Obese class I (30-34)	104 (28.2)	2.47 (0.04)		2.47 (0.04)		2.47 (0.04)	
Obese class II+ (≥35)	70 (19.0)	2.53 (0.05)		2.53 (0.05)		2.53 (0.06)	
Physical activity level (tertiles)							
Light	132 (35.8)	2.50 (0.04)	0.63	2.50 (0.04)	0.63	2.50 (0.04)	0.59
Moderate	145 (39.3)	2.53 (0.04)		2.53 (0.04)		2.53 (0.04)	
Heavy	88 (23.9)	2.47 (0.05)		2.47 (0.05)		2.47 (0.05)	

BMI, body mass index; SE, standard error

[a] Totals may not add up to 100% due to missing responses in <3% of participants.

[b] Those not born & raised in US; N=300.

[c] Variable not assessed prior to 2006; N=333 for 2006-2011 enrollment.
