# Supplementary material for: Oral microbiota reveals signs of acculturation in Mexican American women
Source: PLoS One. 2018 Apr 25;13(4):e0194100. doi: 10.1371/journal.pone.0194100 (PMC5918619; doi:10.1371/journal.pone.0194100)
Supplement: S3 Table — (PDF) [file pone.0194100.s006.pdf]

S3 Table. Differential OTU-level taxa by DMM cluster as determined by LEfSe[a].

OTU	Cluster	Log10 LDA Score	P-value
Unc00gqv	*Streptococcus*	4.9	1.17E-23
Unc01rnu	*Streptococcus*	4.2	1.30E-11
Unc02m1k	*Streptococcus*	4.0	3.64E-06
Unc01c6g	*Fusobacterium*	4.2	2.39E-22
Unc00jei	*Fusobacterium*	3.9	1.14E-12
Unc02sp8	*Fusobacterium*	3.7	1.66E-28
PreInter	*Fusobacterium*	3.7	2.20E-32
Unc86817	*Fusobacterium*	3.7	3.25E-22
UniEub50	*Fusobacterium*	3.5	7.42E-33
Unc28936	*Fusobacterium*	3.4	1.02E-27
Unc19140	*Fusobacterium*	3.4	3.78E-07
UncTr374	*Fusobacterium*	3.3	5.68E-40
TreSpe75	*Fusobacterium*	3.3	2.56E-42
Unc28094	*Fusobacterium*	3.2	1.50E-07
Unid1506	*Fusobacterium*	3.2	4.89E-28
Unc02zcs	*Fusobacterium*	3.2	2.46E-13
FlfAloc4	*Fusobacterium*	3.2	4.80E-44
BAXGenom	*Fusobacterium*	3.2	2.68E-06
TreDent5	*Fusobacterium*	3.2	2.90E-37
Unc01nbb	*Fusobacterium*	3.1	2.27E-23
Unc17528	*Fusobacterium*	3.1	1.19E-18
Unc01qy4	*Fusobacterium*	3.1	2.81E-08
CdlBa105	*Fusobacterium*	3.1	6.11E-08
Unc00hv6	*Fusobacterium*	3.0	2.68E-08
UncPep18	*Fusobacterium*	3.0	3.41E-21
TanFor12	*Fusobacterium*	3.0	2.88E-19
UncPre51	*Fusobacterium*	3.0	9.52E-34
CrfBact8	*Fusobacterium*	3.0	5.78E-06
AclSpec2	*Fusobacterium*	3.0	2.02E-10
Unc61302	*Fusobacterium*	3.0	7.04E-09
UncE4837	*Fusobacterium*	2.9	5.00E-24
BcdGenom	*Fusobacterium*	2.9	5.19E-06
EubSpe41	*Fusobacterium*	2.9	2.03E-07
B9TBact5	*Fusobacterium*	2.9	4.10E-07
PreMarsh	*Fusobacterium*	2.9	1.13E-10
TreSpe28	*Fusobacterium*	2.9	1.22E-36
Unc0027k	*Fusobacterium*	2.9	1.89E-13
Unc006ux	*Fusobacterium*	2.9	1.89E-07
Unc29437	*Fusobacterium*	2.8	4.06E-29
StrAng61	*Fusobacterium*	2.8	1.85E-10
Unc02v33	*Fusobacterium*	2.8	5.11E-06
Unc24661	*Fusobacterium*	2.8	2.96E-11
MpsFauci	*Fusobacterium*	2.8	1.09E-26

TreSpe92	*Fusobacterium*	2.8	4.56E-11
Unc24965	*Fusobacterium*	2.8	2.45E-34
UncTr376	*Fusobacterium*	2.8	1.98E-10
Unc85417	*Fusobacterium*	2.8	6.58E-10
PreGeno3	*Fusobacterium*	2.8	9.53E-06
CddPrev2	*Fusobacterium*	2.8	8.98E-17
UncB9712	*Fusobacterium*	2.8	2.42E-08
Unc00fg2	*Fusobacterium*	2.8	1.23E-17
Unc24653	*Fusobacterium*	2.8	6.50E-10
Unc006v1	*Fusobacterium*	2.8	5.26E-16
Unc0295v	*Fusobacterium*	2.7	5.47E-08
Unc70927	*Fusobacterium*	2.7	1.04E-08
FirmOra5	*Fusobacterium*	2.7	1.88E-07
PptSpec3	*Fusobacterium*	2.7	1.63E-25
TreSpe13	*Fusobacterium*	2.7	8.50E-39
Unc00sox	*Fusobacterium*	2.7	1.07E-18
K10Genom	*Fusobacterium*	2.7	1.08E-15
UncD1684	*Fusobacterium*	2.7	1.11E-11
WlnSpeci	*Fusobacterium*	2.7	3.71E-13
Unc018tj	*Fusobacterium*	2.7	7.18E-09
UncB9875	*Fusobacterium*	2.7	2.67E-12
BacSpec4	*Fusobacterium*	2.7	5.76E-14
CdlBac72	*Fusobacterium*	2.7	1.17E-37
TreLeci3	*Fusobacterium*	2.6	4.76E-22
PreMica2	*Fusobacterium*	2.6	1.24E-13
Unc86775	*Fusobacterium*	2.6	2.27E-18
EubSpe33	*Fusobacterium*	2.6	8.54E-26
Unc83330	*Fusobacterium*	2.6	2.77E-17
EubSaph3	*Fusobacterium*	2.6	1.55E-26
UncJohns	*Fusobacterium*	2.6	4.37E-09
Unc000n0	*Fusobacterium*	2.6	8.23E-17
Unc23873	*Fusobacterium*	2.6	5.24E-07
Unc83810	*Fusobacterium*	2.6	3.15E-18
Unc040gh	*Fusobacterium*	2.6	2.22E-08
Unc00r00	*Fusobacterium*	2.6	1.62E-08
EgrCate9	*Fusobacterium*	2.6	5.31E-10
UncB9956	*Fusobacterium*	2.6	5.23E-13
Unc01npu	*Fusobacterium*	2.6	8.21E-16
PreSpe72	*Fusobacterium*	2.6	2.58E-10
J4KBact6	*Fusobacterium*	2.6	8.83E-28
EubYuri5	*Fusobacterium*	2.6	4.68E-17
UncTr375	*Fusobacterium*	2.6	2.41E-22
SrBacte2	*Fusobacterium*	2.6	4.36E-16
Unc95251	*Fusobacterium*	2.5	2.44E-09
TreSpe87	*Fusobacterium*	2.5	6.75E-09
BactOral	*Fusobacterium*	2.5	6.01E-07
Unc000pl	*Fusobacterium*	2.5	3.99E-25

PccAbsc3	*Fusobacterium*	2.5	1.67E-14
UncP1137	*Fusobacterium*	2.5	4.52E-06
Unc55172	*Fusobacterium*	2.5	7.28E-19
Unc25687	*Prevotella*	4.7	6.59E-30
StrThe42	*Prevotella*	4.4	6.02E-19
Unc0250q	*Prevotella*	4.4	2.54E-27
Unc86427	*Prevotella*	3.7	1.83E-21
UncPrev6	*Prevotella*	3.7	1.65E-30
Unc03why	*Prevotella*	3.6	5.95E-17
Unc86566	*Prevotella*	3.4	1.93E-08
UncO4140	*Prevotella*	3.2	1.65E-20
Unc00roo	*Prevotella*	3.2	8.08E-25
Unc03og4	*Prevotella*	3.2	6.92E-12
Unc02022	*Prevotella*	3.2	2.09E-23
Unc00gj0	*Prevotella*	3.1	9.35E-16
Unid1487	*Prevotella*	3.0	1.06E-08
Unc03jkb	*Prevotella*	2.8	5.20E-19
Unc02f7w	*Prevotella*	2.8	6.28E-18
Unc27997	*Prevotella*	2.8	2.46E-10
LhnGenom	*Prevotella*	2.8	1.90E-13
UncPre78	*Prevotella*	2.7	3.83E-17
Unc037lz	*Prevotella*	2.7	7.62E-15
Unc02zoe	*Prevotella*	2.7	7.38E-11
LprSpec8	*Prevotella*	2.6	1.87E-07
EubSulci	*Prevotella*	2.6	6.74E-16
UncCl508	*Prevotella*	2.6	4.74E-08

[a] LEfSe parameters: minimum LDA=2.5, α=1E-5, one-against-all strategy.
